# Parental Aesthetic Anxiety, Disability Aesthetics, and the Ethics of Surgical Normalization in Congenital Hand Differences

**DOI:** 10.7759/cureus.109577

**Published:** 2026-05-24

**Authors:** Joseph Salem-Hernández, Felix M Rivera-Troia, Leah Cobb, Pablo Marrero-Ortiz, Norman Ramírez

**Affiliations:** 1 Department of Orthopaedic Surgery, Ponce Health Sciences University, Ponce, PRI

**Keywords:** bodily autonomy, congenital hand differences, disability aesthetics, disability studies, parental consent, pediatric ethics, pediatric hand surgery, polydactyly, surgical normalization, syndactyly

## Abstract

Children born with congenital hand differences (syndactyly, fusion of adjacent digits; polydactyly, duplication of digits; radial club hand, partial or complete absence of the radius; and symbrachydactyly, short, webbed, or absent fingers) occupy a unique and underexamined position at the intersection of pediatric surgery and disability studies. While surgical literature has meticulously documented operative techniques, functional outcomes, and complication profiles, it has almost entirely neglected the aesthetic and cultural dimensions of the decision to operate. This narrative review addresses that gap, arguing that surgical intervention for congenital hand differences is implicitly an aesthetic and cultural decision as much as a functional one, and that this dimension demands critical, clinically engaged examination.

Drawing on disability scholars Rosemarie Garland-Thomson and Tobin Siebers, situated within disability studies, a field that examines the social, cultural, and political dimensions of bodily difference, we demonstrate how the normate hand (five-fingered, symmetrical, and fully separated) functions not as a neutral medical standard but as a culturally specific aesthetic ideal. We examine three interlocking dynamics through which this ideal is reproduced: the implicit primacy of aesthetics in surgical decision-making, even when functional impairment is minimal; the role of parental aesthetic anxiety and fear of social stigma in driving demand for early correction; and the frequently overlooked agency and self-concept of children, who research shows often have complex, nuanced, and affirmative relationships to their differences that diverge substantially from parental and medical assumptions.

We further analyze the ethical tensions between parental authority and the child's right to an open future, drawing on parallel debates in intersex surgery to argue that appearance-normalizing procedures performed before the child can meaningfully participate in the decision require stronger justification than current practice typically demands. Psychosocial evidence challenges the foundational assumption that a hand that looks different is inherently stigmatizing: children with hand differences demonstrate robust coping, adaptation, and quality of life, particularly when supported by affirming families and communities.

We propose a disability-affirming framework for clinical practice that prioritizes function over aesthetics, defers elective procedures until the child can participate in the decision, centers the child's own voice in ongoing care, and actively supports parental adjustment to reduce anxiety driven by social stigma rather than the child's wellbeing. The question is not whether children with congenital hand differences can thrive. Evidence shows clearly that they can. The question is whether surgeons, families, and health systems can create the conditions for them to do so on their own terms.

## Introduction and background

The birth of a child with a congenital hand difference, whether syndactyly (fusion of adjacent digits, ranging from simple skin webbing to complex bony union), polydactyly (duplication of digits, most commonly affecting the thumb or small finger), radial club hand (partial or complete absence of the radius, resulting in radial deviation of the wrist), or symbrachydactyly (a spectrum of short, webbed, or absent fingers), initiates a cascade of medical, familial, and cultural responses. Surgical reconstruction is often presented as a primary pathway, justified predominantly by functional goals such as enabling grasp, facilitating bimanual activities, and optimizing hand development [1,2,3]. Yet embedded within these functional goals is a less examined but equally powerful imperative: the aesthetic normalization of the child's body.

Disability studies scholars have long argued that the medical model of disability, which frames bodily difference as pathology requiring correction, obscures the social, cultural, and political dimensions of embodiment [4,5]. Rosemarie Garland-Thomson's concept of the extraordinary body, situated within disability studies and developed to analyze how cultural norms produce hierarchies of bodily value, and Tobin Siebers' framework of disability aesthetics, which fundamentally redefines what counts as beautiful, valuable, and worthy of representation, reveal how bodies that deviate from normative standards become sites of cultural anxiety, spectacle, and corrective intervention [4,5]. In the context of congenital hand differences, these theoretical insights expose a critical gap: surgical literature has meticulously documented techniques and outcomes but has almost entirely ignored the aesthetic and cultural logics that drive the decision to operate in the first place.

This narrative review examines congenital hand differences through the lens of disability aesthetics, asking three questions of mounting urgency: Who decides what a hand should look like? At what age can a child participate meaningfully in that decision? How do parental aesthetic anxiety, surgical norms, and the child's emerging self-concept interact in ways that are both philosophically rich and clinically urgent? By integrating disability studies frameworks with empirical evidence from surgical, ethical, and psychosocial literature, we challenge the presumption that bodily difference requires correction and advocate for a more nuanced, rights-based approach to pediatric surgical decision-making. We do not argue against surgery. We argue for a more deliberate, critically informed approach that is honest about aesthetic motivations, attentive to the child's own perspective, and alert to the long-term implications of decisions made before the child can participate in them.

## Review

Methods

This is a narrative review. Literature was identified through structured searches of PubMed, PsycINFO, and PhilPapers, covering the period 2000-2025, with no language restrictions applied. Search terms included “congenital hand differences,” “syndactyly surgery outcomes,” “polydactyly surgical treatment,” “radial club hand,” “symbrachydactyly,” “disability aesthetics,” “parental decision-making pediatric surgery,” “pediatric surgical consent,” “bodily normalization,” “psychosocial outcomes limb difference,” and “intersex surgery ethics.” Reference lists of retrieved articles were also reviewed for additional relevant sources.

Sources were selected to represent the best available empirical evidence, including surgical outcomes research, psychosocial and qualitative studies, and patient/parent perspective studies, alongside key theoretical and ethical contributions from disability studies and bioethics. Inclusion prioritized studies directly relevant to the review questions: the role of aesthetics in surgical decision-making for congenital hand differences, parental psychological responses to diagnosis, children's self-concept and agency, and ethical frameworks for appearance-normalizing surgery in pediatric populations.

As a narrative review, formal Preferred Reporting Items for Systematic Reviews and Meta-Analyses (PRISMA) protocols, registered searches, risk-of-bias assessments for individual studies, and PRISMA flow diagrams are not required and were not produced. The selection of sources reflects interpretive scholarly judgment aimed at synthesis and critical analysis rather than exhaustive or pooled quantitative summary. Given the heterogeneity of study designs, populations, outcomes, and disciplines represented in the literature, quantitative synthesis was neither feasible nor appropriate. Evidence is synthesized narratively. No statistical software was used, and the review does not require review by a statistician.

Background: Congenital hand differences and surgical norms

Congenital hand differences encompass a heterogeneous group of conditions affecting approximately 1 in 500 to 1 in 2,000 live births [1,2]. The four conditions most relevant to this review are syndactyly, polydactyly, radial club hand, and symbrachydactyly. Syndactyly refers to the fusion of adjacent digits, ranging from simple skin webbing to complex bony union, classified by severity and number of digits involved [6,7]. We acknowledge that even cases classified as simple syndactyly may carry functional implications, including effects on digit growth, dexterity, hygiene, and finger independence. Polydactyly involves the duplication of digits, most commonly affecting the thumb (radial polydactyly) or the small finger (ulnar polydactyly), with varying degrees of skeletal and soft-tissue development [8,9,10]. Radial club hand describes partial or complete absence of the radius, resulting in radial deviation of the wrist and associated thumb hypoplasia [3]. Symbrachydactyly encompasses a spectrum of short, webbed, or absent fingers, often with rudimentary digital nubbins, classified by degree of digital involvement [11,12].

Surgical treatment for congenital hand differences is typically framed around dual goals: functional improvement and aesthetic normalization. For syndactyly, the primary aim is to separate digits to enable independent finger movement and prevent growth disturbances, with surgery typically recommended between six and 18 months of age [6,7]. For polydactyly, reconstruction seeks to create a stable, well-aligned thumb or finger with attention to both functional pinch and cosmetic appearance [10,13]. In symbrachydactyly, more complex reconstructions (including toe-to-hand transfers) aim to create functional digital rays, though outcomes are variable and often prioritize aesthetic appearance over functional gain [11,14]. Across these conditions, surgical literature consistently emphasizes both functional and aesthetic outcomes. One systematic review notes that the goals of surgical treatment are to maximize hand function and aesthetics while minimizing adverse outcomes [15]. Yet the relative weight given to each goal, and the cultural assumptions embedded within aesthetic judgments, remain largely unexamined in clinical discourse.

Surgical timing is typically determined by a combination of anesthetic safety, developmental considerations, and psychosocial factors. For syndactyly, early intervention before 18 months is recommended to prevent growth disturbances and allow for good social adaptation [6,7]. For polydactyly, surgery is often performed between 12 and 18 months, balancing anesthetic risk with the child's lack of awareness regarding aesthetic appearance [10]. For symbrachydactyly, toe-to-hand transfers are ideally performed before one year of age to optimize cortical integration, despite potentially disappointing functional outcomes [12]. Decision-making is typically framed as a collaboration between surgeons and parents, with children often described as not mature enough to express desires [11]. Yet this framing obscures critical questions about whose aesthetic preferences are being served, what assumptions about normalcy are being enacted, and how the child's future autonomy is being protected or foreclosed.

Theoretical foundations: Disability aesthetics and the politics of normalization

Rosemarie Garland-Thomson's concept of the extraordinary body describes how bodies that deviate from cultural norms become marked, spectacularized, and subjected to corrective interventions [4]. In contrast to the normate (the unmarked, idealized body that serves as the implicit standard), extraordinary bodies are perceived as requiring explanation, management, and normalization [16]. This dynamic is not merely descriptive but constitutive: the normate exists only in relation to the extraordinary body, and the cultural work of distinguishing between them produces and reinforces hierarchies of bodily value [4]. In the context of congenital hand differences, the extraordinary body is the hand that deviates from the five-fingered, symmetrical, fully separated norm. Surgical intervention becomes the mechanism through which the extraordinary is rendered ordinary and the child is brought closer to the normate ideal. Yet this process is never neutral: it reflects and reproduces cultural anxieties about difference, disability, and the boundaries of acceptable embodiment [17,18].

Tobin Siebers' framework of disability aesthetics extends this analysis by arguing that disability challenges traditional aesthetic norms and reveals the contingency of beauty standards [5]. Disability aesthetics does not simply advocate for the inclusion of disabled bodies in art and culture; it fundamentally redefines what counts as beautiful, valuable, and worthy of representation. Siebers argues that different bodies require and create new modes of representation and that the aesthetic experience of disability disrupts the presumption that beauty is synonymous with wholeness, symmetry, and able-bodiedness [5]. Applied to congenital hand differences, disability aesthetics invites us to ask: what if a hand with syndactyly, polydactyly, or symbrachydactyly is not a failed approximation of the normate hand, but a different and equally valid form of embodiment? What if the aesthetic judgment that such a hand requires correction is not a medical fact but a cultural preference?

Garland-Thomson's analysis of the stare provides a crucial link between aesthetic norms and social interaction [19]. The stare is the ocular response to visual novelty, a reaction to bodies that surprise, unsettle, or disrupt expectations [20]. For children with visible hand differences, the stare is a ubiquitous experience, one that marks them as extraordinary and subjects them to ongoing social scrutiny [21]. Parental decisions to pursue surgical normalization are often driven by a desire to protect the child from the stare and to minimize the social and psychological burden of being visibly different [22]. Yet disability scholars argue that the stare is not an inevitable response to bodily difference but a learned behavior shaped by cultural norms [23]. The problem is not the extraordinary body but the social conditions that render it extraordinary. Surgical normalization, in this view, shifts the burden of adaptation onto the child rather than challenging the cultural norms that produce aesthetic anxiety in the first place [24].

Aesthetic imperative in surgical decision-making

While surgical literature consistently frames intervention in terms of both function and aesthetics, empirical evidence suggests that aesthetic concerns often drive decision-making in ways that exceed functional considerations. We do not argue that surgical decisions are driven solely by aesthetics, functional improvement, developmental optimization, and prevention of growth disturbance are legitimate and well-documented goals. Our contention is that aesthetic motivations operate in parallel with, and sometimes in excess of, functional ones, and that this dimension deserves explicit acknowledgment in clinical and ethical discussions. For polydactyly, one study notes that aesthetics and appearance were highly valued by patients and parents, especially among females [25]. For syndactyly, another observes that patient and parent preferences concerning aesthetic issues are highlighted as equally important to functional outcomes [26]. In symbrachydactyly, where functional gains from toe-to-hand transfer are often modest, the decision to operate is frequently justified on aesthetic grounds, with parents seeking to create a hand that looks more typical [11].

This aesthetic imperative is particularly evident in cases where functional impairment is minimal or absent. For type B ulnar polydactyly (a small, non-functional extra digit), parents prioritize rapid treatment and express significantly less satisfaction when residual remnants remain visible [27]. For simple syndactyly without bony fusion, where functional limitations are negligible, surgery is still recommended to create a more aesthetic appearance and ensure good social adaptation [7]. These cases reveal that the decision to operate is not primarily about restoring function but about conforming to aesthetic norms.

The aesthetic standard against which congenital hand differences are judged, the five-fingered, symmetrical, fully separated hand, is not a neutral or universal ideal but a culturally specific norm. As Garland-Thomson argues, disability is a cultural attribution of corporeal deviance, not a bodily property [4]. Yet surgical literature rarely interrogates this norm. Instead, it takes the five-fingered hand as the implicit goal, with deviations framed as anomalies or malformations requiring correction [2,3]. This framing obscures the extent to which surgical intervention is an aesthetic and cultural project, one that seeks to align the child's body with prevailing norms rather than to address a medical pathology.

The emphasis on aesthetic outcomes is further evident in how surgical success is measured. For polydactyly, studies report aesthetically satisfactory anatomical reconstruction and parents' satisfaction with appearance as primary outcomes [13]. For syndactyly, scar aesthetics and cosmetic measures are routinely assessed alongside functional measures [28]. For symbrachydactyly, aesthetic outcomes are explicitly prioritized even when functional gains are limited [11]. This focus on aesthetics reflects a broader cultural logic in which the appearance of the hand is understood to have profound psychosocial consequences, a claim that disability studies scholars directly challenge, arguing that psychosocial distress arises not from bodily difference itself but from the social conditions that devalue and marginalize difference [23].

Parental aesthetic anxiety and the burden of choice

Emotional and Social Dimensions of Parental Decision-Making

The birth of a child with a congenital hand difference is often experienced by parents as a crisis, marked by grief, guilt, and anxiety about the child's future [1,29]. Parents describe feeling overwhelmed by the diagnosis and its implications for their child's quality of life, with concerns about social integration, peer acceptance, and the child's ability to lead a typical life [29]. In this context, surgical intervention is often framed as a way to fix the problem, to restore the child to normalcy and to alleviate parental distress [22]. Qualitative research on parents of children with disorders of sex development, a parallel case of appearance-normalizing surgery, found that parents sought surgery to avert teasing and ensure normal daily functioning, though future concerns about identity and relationships persisted [22]. A study of parents of children with congenital hand anomalies found that parents expressed a strong need for support and active participation in surgical decision-making, feeling disappointed when excluded from the process [29]. These findings suggest that parental decisions to pursue surgery are driven not only by concerns about the child's wellbeing but also by parents' own need to manage anxiety and assert control in the face of uncertainty.

Parental concerns about their child's social experiences and future wellbeing are understandable and deeply caring responses that deserve clinical acknowledgment and active support. Parents worry that their child will be stared at, teased, or excluded, and they seek surgical intervention as a way to protect the child from these experiences [22]. As one study notes, parents may not always be aware of the extent to which their own concerns about social reception influence their views on treatment, and their perspectives may not fully reflect those of the child [21]. Another observes that parents' decision-making regarding their child's surgery, and their perception of their child's difference, could exacerbate feelings of uncertainty and anxiety [30]. This dynamic reflects what Garland-Thomson calls the aesthetic anxiety produced by the stare: the discomfort that arises when a body disrupts normative expectations [31]. For parents, the stare is not only a source of concern for the child but also a reflection on their own parenting. Surgical normalization becomes a way to manage this anxiety and align the child's body with the normate ideal.

Yet the desire to normalize the child's body exists in deep tension with parental love and acceptance. One study of parents of children with unilateral forearm agenesis found that parents held a paradoxical view of the child as handicapped yet autonomous, expressing anxiety about social integration while also affirming the child's capabilities [32]. Another study of children with craniofacial conditions found that some children felt pressured by parents to undergo surgery, while others were included in decision-making, with conflicts arising when children did not perceive a need for surgery, viewing their bodies as acceptable [33]. As disability studies scholar Adrienne Asch argues, appearance-altering surgery for children raises profound questions about parental love and the message sent to the child about the acceptability of their body [34]. If the child's body requires surgical correction to be socially acceptable, what does this communicate about the child's inherent worth, and how does this shape their emerging sense of self?

The child's emerging self-concept and agency

Empirical research reveals that children with congenital hand differences have complex and often affirmative relationships to their bodies, relationships that may differ significantly from parental perceptions. One qualitative study found that children as young as five had unique opinions about their congenital hand and upper limb differences, sometimes unknown to parents [21]. Another found that children with congenital limb deformities considered themselves normal and used planned responses as a coping mechanism when faced with unsolicited peer questions [35]. These findings challenge the assumption that children with hand differences inevitably experience their bodies as deficient or stigmatizing. While some children report stress related to hand appearance and social interactions, others describe positive aspects including increased determination and express satisfaction with their bodies [36]. Importantly, children's perspectives evolve over time, shaped by developmental stage, social context, and the messages they receive from parents, peers, and clinicians [37].

The standard recommendation to perform surgery in infancy or early childhood, before the child is aware of their difference or able to express preferences, has profound implications for the child's autonomy and self-concept [7,10]. By intervening before the child can participate in the decision, parents and surgeons foreclose the possibility that the child might choose to live with their difference, develop a positive relationship to their body, or reject the normate ideal altogether. As one ethical analysis notes, traditional consent may be insufficient for appearance-normalizing surgeries due to their elective nature and alteration of natural identity [38]. Another argues that decisions should not rest on psychosocial discomfort, but on the child's developmental capacity for decision-making [39]. These critiques highlight the tension between parental authority and the child's right to an open future, the right articulated by philosopher Joel Feinberg to make decisions about their own body and identity as they mature [40].

Studies of adolescents and adults who underwent surgery as children reveal a range of perspectives, some affirming the decision and others expressing regret or ambivalence. One study of adolescents with neonatal brachial plexus palsy found that as adolescents aged, their desire for independent communication and influence on medical decisions increased, leading to shared or autonomous decision-making [25]. A study of adolescents with congenital limb reduction deficiency found that childhood treatment shaped their current sense of self, contributing to creating opportunities, choosing one's own path, and belonging in a context [37]. However, parallel evidence from the intersex literature is more troubling. Research on individuals who underwent early genital surgery found that many experienced pain, humiliation, and poor functional outcomes, and reported a perceived violation of their human rights [41]. Studies of intersex adults found that those provided with surgical approximations of typical anatomy later wished they had been accepted as different rather than subjected to surgical normalization [42]. These testimonies underscore the risk that early surgical intervention, driven by parental and medical aesthetic preferences, may not align with the child's own values as they mature.

Ethical tensions: Rights, autonomy, and medical paternalism

Disability rights frameworks fundamentally challenge the presumption that bodily difference requires correction. As one human rights analysis argues, early, irreversible, and normalizing procedures without consent violate human rights to health, body integrity, autonomy, and reproductive rights [43]. These critiques rest on the principle that children have a right to bodily integrity and that interventions aimed at aesthetic normalization rather than addressing functional impairment or medical necessity, cannot be justified on purely paternalistic grounds. The critique of normalization has been most fully developed in the context of intersex surgery, where international human rights bodies have called for a moratorium on non-urgent, appearance-normalizing procedures [44,45]. While congenital hand differences differ from intersex conditions in important ways, including their anatomical scope, functional implications, hormonal dimensions, and developmental trajectories, the underlying ethical logic is similar: interventions that alter the child's body to conform to aesthetic norms, without the child's consent, raise profound questions about autonomy, bodily integrity, and the right to self-determination [46]. We do not argue that these conditions are equivalent; rather, we draw on the intersex debate as an ethical parallel whose underlying principles are transferable in their logic even where the medical specifics differ substantially.

The tension between parental authority and child autonomy is central to debates about appearance-normalizing surgery. Parents are typically granted broad discretion to make medical decisions on behalf of their children, based on the principle that they are best positioned to act in the child's best interest [39]. Yet this discretion is not unlimited: it is constrained by the requirement that interventions serve the child's medical needs, not merely parental preferences or social norms [38]. In the case of congenital hand differences, the boundary between medical necessity and aesthetic preference is frequently blurred. For conditions like simple syndactyly or type B ulnar polydactyly, where functional impairment is minimal, the decision to operate is driven primarily by aesthetic concerns [7,27]. In these cases, the justification for parental authority is weaker, and the argument for deferring intervention until the child can participate in the decision becomes substantially stronger [47,48]. As one ethical analysis argues, protecting the child's best interest requires seeking perspectives from individuals living with the trait to enable truly informed choices [38]. Another states that children have a right to know about their bodies and the value of individuality and diversity over humiliation and shaming [39].

Surgical norms themselves reflect a form of medical paternalism in which the presumption that difference requires correction is rarely questioned. In the context of congenital hand differences, this paternalism is evident in the routine recommendation for early surgical intervention, the emphasis on aesthetic outcomes, and the framing of non-intervention as a failure to act in the child's best interest [1]. Yet this framing obscures alternative possibilities: that the child might adapt functionally without surgery, might develop a positive relationship to their difference, or that the psychosocial benefits of surgery might be overstated [49]. Disability rights frameworks challenge this paternalism by insisting that the decision to intervene be grounded in the child's own values and preferences, not in normative assumptions about what bodies should look like [43].

Toward a disability-affirming approach to congenital hand differences

A disability-affirming approach to congenital hand differences begins by reframing the goals of care. Rather than assuming that the primary goal is to make the child's hand look normal, care should be oriented toward supporting the child's functional adaptation, psychosocial wellbeing, and emerging autonomy [1]. The first shift concerns the primacy of function over aesthetics. Interventions should be justified primarily on the basis of functional improvement, not aesthetic normalization. For conditions such as simple syndactyly or type B ulnar polydactyly, where functional impairment is minimal or absent, the case for early surgical intervention requires more rigorous justification than current practice typically demands [7,27]. The second shift concerns the timing of elective procedures. Where surgery is primarily aesthetic in its motivation, decision-making should be deferred until the child is old enough to participate meaningfully, thereby preserving the child's autonomy and allowing them to develop their own relationship to their body before irreversible decisions are made on their behalf [47,48]. The third shift concerns how clinicians and families communicate about difference itself. Parents and clinicians should convey to children that their bodies are valuable and acceptable as they are, and that surgical intervention is one option among many and not a necessity and not a measure of the child's worth or belonging [39].

Supporting Parents in Adjusting to Their Child's Difference

A disability-affirming approach also requires actively supporting parents in adjusting to their child's difference. Three practical strategies support this goal. Connecting parents with other families of children with hand differences, and with adults who have hand differences, helps parents encounter alternative narratives and reduces the sense of urgency around surgical intervention [50]. Rather than accepting stigma as an immovable social fact, parents and clinicians can actively challenge the norms that produce aesthetic anxiety, educating peers, teachers, and community members about bodily diversity and modeling constructive responses to difference [23]. And parents should be directly informed that psychosocial wellbeing is not determined by the appearance of the hand but by the child's sense of acceptance, competence, and belonging, a claim supported by evidence showing that children with hand differences cope and adapt well, with overall quality of life remaining favorable regardless of whether the hand conforms to normative appearance [49].

Centering the Child's Voice

Finally, a disability-affirming approach centers the child's voice in decision-making at every stage. In practice, this means that clinicians should routinely assess the child's own views about their hand, their experiences of social interaction, and their preferences regarding intervention, recognizing that children as young as five hold unique opinions about their differences that are sometimes entirely unknown to their parents [21]. As the child matures, they should be increasingly involved in decisions about their care; for adolescents, this may mean shared or fully autonomous decision-making, with the child's stated preferences carrying significant clinical weight [25]. And if a child expresses satisfaction with their body and does not wish to pursue surgery, that preference deserves genuine respect, even when it diverges from parental expectations or medical recommendations [33].

The ethical debates surrounding intersex surgery offer important parallel lessons. International human rights bodies have increasingly called for a moratorium on non-urgent, appearance-normalizing surgeries for intersex children, arguing that such interventions violate the child's right to bodily integrity and self-determination [44,45]. While congenital hand differences differ from intersex conditions in meaningful ways, the underlying ethical principles are transferable: interventions that alter a child's body to conform to aesthetic norms, without the child's consent, require strong justification and ongoing critical scrutiny [46]. Many intersex adults report that they wished they had been accepted as different rather than subjected to normalization, and that early surgery caused lasting physical and psychological harm [51]. These testimonies underscore the importance of deferring elective procedures until the child can participate in the decision, and of challenging the presumption that difference requires correction.

## Conclusions

This review has argued that surgical intervention for congenital hand differences is implicitly an aesthetic and cultural decision as much as a functional one, and that this dimension demands urgent critical examination. Drawing on Rosemarie Garland-Thomson and Tobin Siebers, we have shown how the decision to operate reflects and reproduces cultural norms about what bodies should look like, who holds the authority to make aesthetic judgments, and what it means to live with bodily difference. A disability-affirming approach requires reframing the goals of care, supporting parental adjustment, and centering the child's voice at every stage of decision-making. It demands that the primacy of function over aesthetics be made explicit, that elective appearance-normalizing procedures be deferred until the child can participate meaningfully, and that parents be supported in recognizing that psychosocial wellbeing is determined not by the appearance of the hand but by the child's sense of acceptance, competence, and belonging.

As disability rights frameworks increasingly challenge the normalization paradigm, pediatric hand surgery has an opportunity to lead by example. The ethical debates surrounding intersex surgery offer transferable lessons: interventions that alter a child's body to conform to aesthetic norms, without the child's consent, require strong justification and ongoing critical scrutiny. The question is not whether children with congenital hand differences can thrive. Evidence shows clearly that they can and do. The question is whether clinicians, families, and health systems can create the conditions for them to do so on their own terms.
